# Reducing Domestic Wood Burning through Voluntary Air Quality Alerts: An IBM-WASH Evaluation of a Pilot Intervention in Wales

**DOI:** 10.1007/s00267-026-02463-8

**Published:** 2026-04-22

**Authors:** James Heydon, Menna Price, Ian Walker, Paul Lewis, Rohit Chakraborty, Caitlin Bunce, Kori Sunter

**Affiliations:** 1 University of Nottingham, Nottingham, UK; 2 Swansea University, Swansea, UK; 3 Swansea University, Raven Delta Group, Swansea, UK; 4 UK Health Security Agency, London, UK

**Keywords:** Air quality, Air pollution, Domestic burning, Voluntary regulation, IBM-WASH

## Abstract

Burning solid fuels for home heating is a major source of fine particulate matter (PM2.5) and associated health risks across Europe. This article reports findings from a pilot study of a voluntary ‘burn alert’ system in Swansea, Wales, aimed at reducing domestic burning emissions. The system combined dense air quality (AQ) monitoring, postcode-level PM2.5 data, and behaviorally informed SMS/email alerts discouraging burning during poor air quality episodes. A sequential mixed-methods design included pre- and post-intervention surveys (*n* = 49) and follow-up interviews (*n* = 14). Over four weeks of use, 84% of participants (*n* = 41) reported avoiding burning at least once on receipt of an alert. Among those providing quantitative estimates of burning behavior (*n* = 47), 606 total hours of avoided burning were reported. AQ literacy also increased significantly among participants completing both surveys (paired t-test: t(40) = 2.04, *p* <0.05), with mean scores rising from 7.9 to 8.6 (out of 12). Using the Integrated Behavioral Model for Water, Sanitation and Hygiene (IBM-WASH), qualitative findings indicate that engagement was shaped by contextual factors (including energy costs and regulatory perceptions), psychosocial factors (including trust, self-efficacy and health concerns), and technological factors (including alert timing and perceived sensor proximity). Although recruitment was low relative to the wider burning population, the findings indicate that voluntary, hyper-local alert systems may support behavior change and improvements in AQ literacy among engaged users. Meaningful population-level emission reductions are therefore likely to depend on integrating such systems within broader regulatory and public information strategies, alongside measures to address household energy pressures.

## Introduction and Background

Burning solid fuels for home heating is a major source of fine particulate matter (PM2.5) emissions and a growing public health concern. Across Europe, over half of PM2.5 emissions originate from domestic burning (European Environment Agency, 2022), with residential biofuel use increasing by 82% since 1990 (Kangas et al., 2024: p1490). Exposure to these emissions is associated with a wide range of adverse health outcomes across the life course, including respiratory, cardiovascular and neurodegenerative conditions (Garcia et al. 2023). Current evidence indicates no known threshold below which PM2.5 exposure does not pose health risks, with harmful effects observed even at low concentrations (Yu et al., 2024). As a result, the combined health-related social costs of domestic burning across the EU and UK are estimated at around €29 billion per year (Kortekand et al., 2022: 5)

In this context, domestic burning has become an increasingly salient policy issue. Following the introduction of stove emission limits under the EcoDesign Directive 2009/125/EC, the European Commission is considering stricter regulations from 2027 onwards (Kurmayer, 2025). In the interim, many European authorities are seeking more restrictive controls (Eurocities, 2025), contributing to an uneven and evolving regulatory landscape.

One possible factor contributing to the rise in domestic burning is low air quality (AQ) literacy among the public (Department for Environment, Food and Rural Affairs [Defra], 2025a; 2024). AQ literacy is a multifaceted construct that describes people’s understanding of how air pollution is caused, the subtypes that exist, the risks these create, and how their effects can be mitigated (Sunter et al., forthcoming). Across the EU and UK, public awareness of AQ problems remains limited and has changed little over the last decade (Defra 2025a; Louro et al., 2024; EC 2022). On domestic burning specifically, users are often unaware of its contribution to PM2.5 (Defra 2024), the regulations governing use (Casey et al., 2023), or that emissions occur indoors as well as outdoors (Heydon & Chakraborty, 2022).

This has resulted in the intersection of three critical issues: there is (1) a growing public health risk unfolding within (2) an inconsistent policy landscape, hindered by (3) low public awareness that domestic burning is a problem. In response, this study trialed a pilot, exploratory air quality alert system designed to discourage domestic burning during periods of locally elevated PM2.5. This early-stage trial assessed whether, and how, the system influenced burning behavior and AQ literacy among users. To explain patterns of engagement and response, the Integrated Behavioral Model for Water, Sanitation and Hygiene (IBM-WASH) was applied as an analytical framework (Dreibelbis et al., 2013), with Wales serving as the study location.

## The Limits Of Domestic Burning Regulations in the UK and Wales

Domestic burning is a dominant source of particulate matter across the UK but is particularly significant in Wales, where it contributes over one third of annual PM2.5 emissions (National Atmospheric Emissions Inventory, 2024: 41). In response, the Welsh Government has introduced a series of AQ policies, including stricter PM2.5 targets and restrictions on the sale of heavily polluting domestic fuels, under the Environment (Air Quality and Soundscapes (Wales)) Act 2024. The Act also strengthens local authorities’ powers to issue civil penalties for smoke emissions within Smoke Control Areas (SCAs).

SCAs remain the primary regulatory mechanism for domestic burning across the UK. They designate specific geographic zones where use of ‘non-authorized’ fuels or appliances is prohibited on the basis that these are more likely to produce visible smoke (Davies, 2025). Authorized appliances and fuels are defined by Defra exemption lists, which specify those permitted under current SCA rules (see Defra, 2025b; 2025c). However, despite recent amendments, SCAs exhibit multiple limitations. First, their geographic coverage is sparse and uneven, leaving large areas of urban and rural Wales outside their remit (Welsh Government, 2025). Second, enforcement of SCA rules is stymied by resource constraints, low public awareness, and practical difficulties evidencing breaches (Heydon, 2023). Finally, even when used as intended, authorized appliances emit substantial quantities of PM2.5. Ecodesign stoves, which now account for a substantial proportion of stoves sold in the UK (Stove Industry Association, 2023), emit over three hundred times the PM2.5 of a gas-fired boiler (Chief Medical Officer, 2022: xiv). Taken together, rising stove uptake risks undermining per-appliance efficiency gains and further limiting the effectiveness of the SCA regime.

Burn alerts offer a way to address several of these regulatory limitations. Rather than relying on the smoke-based, area-bound enforcement of SCAs, burn alerts use behaviorally informed messaging to encourage voluntary compliance, discourage burning during periods of elevated PM2.5, and improve AQ literacy among users. By shifting the emphasis from compliance monitoring to informed decision-making, such systems aim to reduce emissions without relying on statutory change or more punitive regulations.

## Study Contributions

This study makes empirical, theoretical and policy contributions. Empirically, it reports the first burn alert trial to combine a bespoke, high-density official sensor network with real-time, postcode level PM2.5 data to deliver street-specific ‘push’ alerts via SMS and email. This level of spatial granularity and personalization extends the functionality of existing alert systems, which typically rely on regional averages or require users to manually access information (Heydon et al., 2024). The study also provides the first empirical assessment of how engagement with burn alerts affects AQ literacy, which may be an important precursor to sustained behavior change (Mogles et al., 2017). Taken together, these findings add to emerging evidence on the potential role of local, voluntary AQ interventions in shaping behavior and improving public understandings in real-world settings.

Theoretically, the study extends the reach of the IBM-WASH (Dreibelbis et al., 2013). While the framework was originally developed to explain technology uptake in low-income contexts (Paasche et al., 2022; Tamene & Afework, 2021), it has only recently been applied to high-income settings (Jewitt et al., 2023; Kostas-Polston et al., 2022) and AQ issues (Clasen & Smith, 2019; Jewitt et al., 2022). By applying IBM-WASH to a voluntary, digitally mediated AQ alert system in Wales, this study extends the framework to a new behavioral domain and socio-economic context. In doing so, it demonstrates how the model can be used to explain uneven engagement, partial compliance, and non-participation in voluntary interventions.

The findings also have policy relevance. In Wales, as elsewhere in the UK, SCA rules continue to focus primarily on visible smoke. This means PM2.5 is addressed only indirectly under legislation such as the Environment (Air Quality and Soundscapes (Wales)) Act 2024. Burn alerts invert this approach by directly targeting particulate matter-producing behavior, offering a mechanism aligned with the Act’s objective of reducing population exposure to PM2.5 by 2030. The findings suggest a voluntary system alone is unlikely to achieve population-level reductions without wider public awareness and uptake. However, improvements in AQ literacy suggest they may play a role in building the awareness and engagement on which the effectiveness of such systems depend.

In this context, burn alerts could complement existing statutory mechanisms by providing a rapid, relatively low-cost addition to current monitoring and regulatory approaches. Although enforced alert regimes exist elsewhere (see Heydon et al., 2024), introducing coercive measures without public support may be politically contentious, particularly given the role of energy costs in driving use (Price-Allison et al., 2023). A voluntary system may therefore represent a more workable near-term step, while retaining the potential for later integration with more stringent SCA-based regulation if needed (see Heydon et al., 2024).

## The IBM-WASH Analytical Framework

The IBM-WASH framework for understanding user behavior was used for several reasons. First, the previous UK-wide pilot of a less advanced burn alert system showed high usability and positive behavior change among users (Heydon et al., 2024). However, it also identified a variety of factors beyond the system itself that affected engagement. This highlighted the need for an analytical lens sensitive to a wide range of behavioral influences on intervention use. The model identifies these across five ‘levels’ and three ‘dimensions’ (see Table 1). The three dimensions capture ‘contextual’ influences, which speak to the structural features of a setting, ‘psychosocial’ factors, that speak to the opportunity, ability and motivational determinants of behavior change, and ‘technology’, which focuses on characteristics of the intervention being introduced. Factors within each dimension can exert influence at four analytical ‘levels’, with a fifth ‘habitual’ level being ‘nested’ within the individual but represented separately (Dreibelbis et al., 2013: 6).

**Table 1:** The IBM-WASH Model

Levels	Contextual factors	Psychosocial factors	Technology factors
Societal/Structural	Policy and regulations, climate and geography	Leadership/advocacy, cultural identity	Manufacturing, financing, and distribution of the product; current and past national policies and promotion of products
Community	Access to markets, access to resources, built and physical environment	Shared values, collective efficacy, social integration, stigma	Location, access, availability, individual vs. collective ownership/access, and maintenance of the product
Interpersonal/Household	Roles and responsibilities, household structure, division of labor, available space	Injunctive norms, descriptive norms, aspirations, shame, nurture	Sharing of access to product, modeling/demonstration of use of product
Individual	Wealth, age, education, gender, livelihoods/employment	Self-efficacy, knowledge, disgust, perceived threat	Perceived cost, value, convenience, and other strengths and weaknesses of the product
Habitual	Favorable environment for habit formation, opportunity for and barriers to repetition of behavior	Existing habits, outcome expectations	Ease/effectiveness of routine use of product

This model readily transfers to domestic burning. Clasen and Smith’s (2019) call for ‘the “A” in WASH [to] stand for Air’ was based on the fundamental similarities between WASH and household air polluting behaviors, the latter of which extend to domestic combustion. Like cookstoves (Jewitt et al., 2022), latrines (Tamene and Afework, 2021), handwashing stations (Hulland et al., 2013), and energy consumption (Walker & Hope, 2020), burning is woven into daily routines, motivated by overlapping health and non-health motivations (Kantar, 2020; Cupples et al., 2007), and shaped by insufficient policy responses (Heydon, 2023). As with WASH behaviors, domestic burning is therefore best understood as a routine practice embedded within wider contexts.

Domestic burning also produces diffuse, community-level effects that impose disproportionate health burdens on vulnerable groups, consistent with patterns identified in WASH research (Paasche et al., 2022). Across England and Wales, urban areas average 62.5 stoves per km^2^, more than twice the rural density of 30.8 km^2^ (Horsfall et al., 2025). This clustering intensifies exposure. While Wales-specific data is sparse, this clustering has been widely evidenced elsewhere (Kangas et al., 2024; Casey et al., 2021; Kukkonen et al., 2020). Much like fecal or water contaminants in dense WASH settings (Clasen and Smith, 2019), this also means that individual shifts to cleaner practices are more effective when occurring at scale. Taken together, these features illustrate the value of IBM-WASH for not only understanding the intersecting factors shaping engagement with burn alert messaging, but also providing targeted suggestions for future improvement.

## Intervention Design

**Fig. 1:**
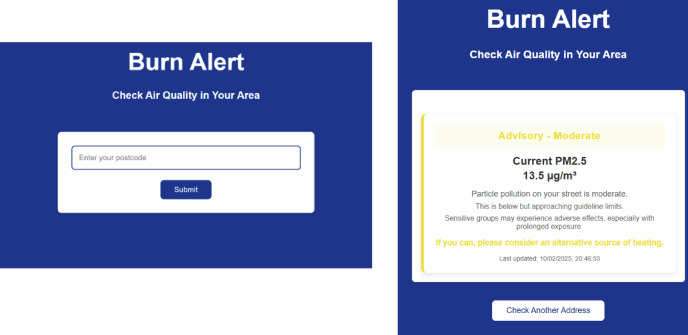
Website Home and Example Alert Pages

Existing communications research evidences the importance of locality in perceptions of AQ (Riley et al., 2021). Accordingly, participants received notifications via SMS and/or email when AQ at their home postcode crossed a given threshold (see Section 3.1). This was accompanied by a website where users could input their postcode and receive up-to-date AQ information at any time. Figure 1 illustrates the website and alert messaging, the latter of which was displayed on both the webpage and in the emails. These were available in both English and Welsh languages.

### Alert AQ Thresholds

A set of bespoke thresholds based on one-hour average PM2.5 concentrations were developed to structure the alerts in response to three inter-related challenges. First, existing thresholds in the UK’s Daily Air Quality Index (DAQI) rely on 24-h averages that smooth out the short-term pollution spikes associated with domestic burning. This made them less appropriate for alerting people to episodic, highly-localized emissions. Second, DAQI thresholds are underpinned by a relatively sparse official monitoring network designed to characterize regional trends rather than local-level dynamics (Louro et al., 2024: 14). This can elicit concerns among users about the credibility of postcode-level alerts based on limited sensor coverage (Heydon et al., 2024). Third, any alert system must balance sensitivity against the risk of both ‘alert fatigue’, where overly-frequent warnings undermine engagement, and ‘green fatigue’, where prolonged periods of unchanging messages reduce responsiveness.

The threshold structure and alert design were developed with these considerations in mind. One-hour PM2.5 concentrations were used to detect short-term emission events in near real-time. This was supported by the installation of eighteen additional monitors to provide dense, hyper-local coverage across Swansea. Threshold bands were informed by exploratory analysis of three years of historical PM2.5 data across Swansea, and by evidence that no exposure threshold has been identified below which adverse health effects do not occur (Committee on the Medical Effects of Air Pollutants, 2022). Thresholds were therefore set to produce perceptible changes in alert status without generating excessive alert frequency, with the intention of primarily supporting behavioral decision-making. Multiple color gradations informed by climate changes communications research (McQuaid et al., 2024), were organized within a three-part alert hierarchy, making changes in air quality more visible while maintaining a simple design.

### System

The burn alert was accompanied by an animation outlining the system rationale, guidance on how to use it, and brief information on the sensor network underpinning the alerts (see Swansea Burn Alert, 2024). This was intended to enhance AQ literacy among stove users prior to their engagement with the system, aligning with established research in health and environmental psychology highlighting the importance of improving AQ awareness (McCarron et al., 2023). The animation was careful not to demonize burning practices and included references for all information provided, with sources drawn from those deemed trustworthy (see Wood et al., 2023).

AQ data was drawn from Swansea City Council’s expanded monitoring network. This included one reference-grade station, five Zephyr units, and eighteen BettAir sensors reporting at 15–60-min intervals. Both the Zephyr and BettAir sensors use optical particle counting (light scattering) for PM2.5 measurement, with manufacturer-reported R² > 0.8 against reference methods under field conditions. These latter sensors were installed to increase spatial density relative to the official network and capture local variation in AQ. Sensors were initially deployed to target specific postcodes, but some were repositioned during the trial after this approach proved impractical in certain locations. For this reason, exact sensor locations – such as using a fixed distribution map – were not provided to participants.

Postcode-level estimates were generated for every participant and messages auto dispatched using the UK Government’s email/SMS alert service in Welsh or English, depending on participant preference. Alerts followed the FEMA message structure outlined in Table 2. The messaging for this project used the four-part framing system known as FEMA: Fact, Evaluation, Motivation, Action. Previously deployed in behavior change research to reduce domestic energy consumption and improve layperson energy literacy (Mogles et al., 2017), Table 2 illustrates the application of this structure to determine the specific wording of messages issued at each alert level. Other research-informed features of message source, content, channel and recipient designed into the prior system were continued here (see Heydon et al., 2024).

**Table 2:**
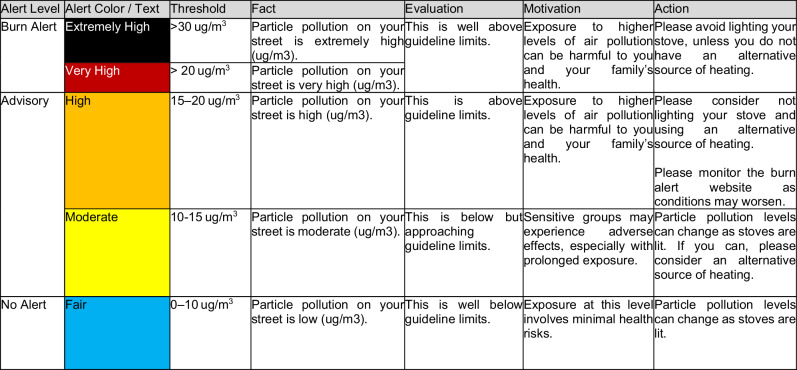
Burn Alert Thresholds and Messaging Structure

## Methods

The study was conducted in Swansea, a coastal city in South Wales with an estimated population of approximately 238,000 (Swansea Council, 2024). Swansea contains a mix of urban, suburban and semi-rural neighborhoods, with a small number of Smoke Control Areas (Welsh Government, 2025). The city was selected due to the presence of an expanded local authority monitoring network and prior evidence of concentrated domestic burning emissions (Lewis, 2023). The intervention was implemented across multiple Swansea postcodes between December 2024 and March 2025.

Between December 12, 2024, and March 31, 2025, 49 stove and fireplace users across Swansea were recruited to test the system over four weeks. Participation was limited to adults (18 + ), residents with Swansea postcodes, and those burning solid fuel as a secondary heat source. This latter exclusion criterion served to minimize the risk of alerts discouraging those without alternative heating from warming their homes (approx. 5% of stove users; Kantar 2020: 11).

The study was originally designed as a quasi-experimental intervention comparing area-level outcomes between intervention and matched control sites. Bilingual (Welsh and English) letters and leaflets were distributed to over 3,000 households across 24 areas in Swansea, selected for their high burner concentrations and levels of deprivation (see Lewis, 2023). However, the exceptionally low response rate made this comparative design unfeasible. The study was therefore adapted to a broader exploratory design using purposive sampling to recruit participants through social media, council and university email lists. This shift limited the ability to conduct controlled comparisons or draw population-level inferences. However, it did provide insight into the likely uptake of a voluntary alert system introduced without an accompanying public information campaign.

A sequential mixed methods research design was used to evaluate the effects of the burn alert system on stove user behavior and AQ literacy. This involved three components administered in turn. First, a structured pre-intervention survey (Pre-S) was used to capture baseline characteristics, AQ literacy and pre-existing burning behaviors and perspectives (*n* = 49). Secondly, after four weeks of burn alert use, a post-intervention survey (Po-S) with the same participants was used to compare pre- and post-survey responses, and capture user experiences. AQ literacy was assessed using twelve multiple-choice items adapted from the Swansea Air Quality Literacy Scale (SAQLS; Sunter et al., forthcoming). The full SAQLS consists of twenty items measuring a broad range of AQ-related knowledge (e.g. engine idling). Here, analysis focused on the subset of items directly relevant to domestic wood burning. Similarly, the 10-item Positive System Usability Scale (SUS) was used in the Po-S to assess usability (Hyzy et al., 2022). The pre-intervention and post-intervention surveys are provided in Supplementary Information I and II. Finally, follow-up qualitative online interviews (Int) with a sub-sample (*n* = 14) were used to explore these effects and experiences in greater detail.

Participant identifiers used in the analysis combine a unique participant number with engagement level and data source. Engagement levels are denoted as High, Moderate, or Low/No, based on self-reported compliance with burn alert advice (see Section 5.1). ‘Pre-S’ denotes data drawn from the pre-intervention survey, ‘Po-S’ from the post-intervention counterpart, and ‘Int’ indicates data from follow-up interviews. Qualtrics was used to administer the surveys. SPSS and Microsoft Excel were used to analyze quantitative data, while NVivo was used for qualitative analysis. Interviews were conducted and transcribed using Microsoft Teams.

Thematic analysis followed Braun and Clarke’s (2006) six-phase framework, using a hybrid deductive-inductive approach (Fereday and Muir-Cochrane, 2006). Initial coding was conducted by one researcher. This was deductively informed by the IBM-WASH framework while also allowing for the creation of themes inductively. Coding proceeded iteratively through repeated reading of transcripts, comparison across responses, and refinement of themes. Although coding was undertaken by a single researcher, theme development and supporting extracts were reviewed and discussed in detail with two co-authors. These discussions involved reviewing coded extracts, clarifying theme boundaries and refining interpretations. Consistent with reflexive thematic analysis, inter-rater reliability statistics were not calculated. Open text responses were analyzed in relation to their corresponding closed survey items (see Rouder et al., 2021). Quotations exemplify analytically derived themes rather than representing the full distribution of responses, consistent with established qualitative research practice.

Ethical approval was granted by the University of Nottingham’s Ethics Sub-Committee (Ref: 97519) and Swansea University’s Ethics Committee (Ref: 10523). All participants provided informed consent prior to participation. Participation was voluntary, with those taking part informed of their right to withdraw at any time without penalty. Survey and interview data were anonymized, stored securely, and handled in accordance with UK data protection legislation. No personal identifying information is reported in this article. All participants who completed both surveys were entered into a prize draw to win one of twenty £20 shopping vouchers.

## Results

Forty-nine stove users participated in the trial. Sample characteristics can be viewed in Table 3. The ethnic composition was broadly reflective of Swansea’s population, though participants were more highly educated than the local average (Swansea Council, 2024). As the sample was purposively restricted to domestic burners rather than the wider population, population-level comparisons should be understood as providing context only. There is no baseline against which to assess the socio-demographic representativeness of stove users in Swansea.

**Table 3:** Sample Characteristics (*n* = 49)

Characteristic	Domestic Burners
Age	Mean = 50.2 (SD 12.1), range = 25 to 74 years
Gender	65% Female; 35% Male
Ethnicity	94% White; 4% Mixed; 2% Other
Children ( < 18) in the home	44% Yes; 56% No
Respiratory illness in household	22% Yes; 78% No
Homeowner	96% Yes; 4% No
In a smoke control area?	2% Yes; 51% No; 47% I don’t know
Alert medium	50% SMS only; 27% SMS & Email; 23% Email only;

### Behavioral and AQ Literacy Outcomes

Over four weeks, 84% (41 of 49) of participants avoided burning at least once on receipt of an alert. Among these, participants reported a total of 606.25 h of avoided burning. This equated to an average reduction of 3.7 h per household per week. In the pre-intervention survey, participants reported burning for an average of 9.8 h per week. Compared with the avoided burning reported in the post-intervention survey, this indicates an average reduction of around 38%. Using participants’ self-reported avoided burning hours and the number of alerts they reported acting on, each acted-on alert corresponded to approximately 3.5 h of avoided burning (IQR: 3–5, median: 4). 11 of 41 participants (27%) who avoided burning at least once also reported shortening the duration of burns when they did light. These estimates assume that avoided or shortened burning episodes would otherwise have occurred in line with participants’ typical burning patterns, as reported in the pre-intervention survey. Using only respondents who fully completed both relevant survey sections (41 of 49), AQ literacy also improved over the trial period. The mean pre-system score was 7.91/12 (SD = 1.41), and the post-system score increased to *M* = 8.56/12 (SD = 1.45), a mean change of *M* = 0.65. A paired t-test indicated a statistically significant increase from pre- to post-use (t(40) = 2.04, *p* <0.05).

While these results show reductions in burning and improvements in AQ literacy, the outcomes were unevenly distributed across participants. For analytic purposes, participants were grouped according to self-reported engagement with alert advice. High Engagement households (24 of 49; 49%), defined as those who reported ‘always’ or ‘often’ following alert advice, prevented 363.75 h of burning in total (60% of all avoided burning). Moderate Engagement households (‘sometimes’ following advice, 11 of 49; 22%) prevented a further 222 h (36.6%), while Low/No Engagement households (‘rarely’ or ‘never’ following alert advice, 14 of 49; 29%) contributed only 20.5 h (3.4%). This indicates that while the system achieved positive changes overall, the degree of compliance varied considerably across households. The following section explores this variation using the IBM-WASH model to analyze the factors shaping participant willingness to act on alerts.

### IBM-WASH Analysis of Intervention Engagement

Table 4 illustrates that engagement with alerts was shaped by a combination of contextual, psychosocial and technological factors operating across levels. Contextual influences, such as high energy prices, sunk installation costs, and the availability of cheap or free fuel was seen to constrain compliance, even where participants accepted the rationale for the alerts. By contrast, psychosocial influences, especially health concerns for vulnerable household members and neighbors, supported higher levels of engagement and helped frame compliance as socially responsible behavior. Yet these same motivations were tempered by wider perceptions of fairness and trust. Several participants contrasted their own burning with other sources, such as traffic and industry, and interpreted ‘authorized’ appliance labels as evidence of environmentally legitimate practices, reducing their perceived need to follow alerts.

**Table 4:** Summary of Contextual, Psychosocial and Technology Influences on Burn Alert Engagement

Level	Contextual Factors	Psychosocial Factors	Technological Factors
Societal/Structural	Cost of living, energy prices, smoke control legislation, ‘authorized’ appliance and burning labels, temperature	Presence of other polluters, social acceptability of burning, trust in authorities, perceived regulatory unfairness/fear of prohibition	Authorized appliance & fuel specifications
Community	Local availability of cheap/free fuel	Health concerns for neighbors/proximate others, sense of social responsibility	Location/density/proximity of sensor network
Interpersonal/Household	Access to viable heating alternatives, ongoing and sunk-cost considerations	Health concerns relating to those in the household (some with existing vulnerabilities)	Alert timings and household routines
Individual/Habitual	Frequency of burner use, cost perceptions associated with fuel stockpiling	Comfort and aesthetics, sense of self-efficacy, environmental discounting, trust in accuracy of data	System usability, alert fatigue

Technological factors influenced these responses but rarely determined them outright. While the system was rated as highly usable, alert frequency, message timing, and uncertainty over sensor placement affected trust in the data. For many, alerts conflicted with routine lighting practices, and doubts about monitor locations prompted reliance on sensory cues instead of the system. These findings show that compliance is not driven solely by system design, but by the intersection of system features with household routines (as in Walker & Hope, 2020), financial pressures and information environments. Voluntary engagement with alert messaging is therefore contingent on broader socio-economic contexts as well as the technical features of the intervention itself.

Although the IBM-WASH model helped to sensitize analysis to these different dimensions, participant accounts did not map neatly onto its discrete categories. Instead, experiences clustered around cross-cutting themes that reflected interactions between levels and factors. For this reason, the findings are presented through four interacting themes that together explain patterns of differential engagement, rather than being organized strictly by the framework. This is also consistent with the ‘hybrid’ coding approach adopted (see Section 4).

#### System Usability and Alert Features

**Fig. 2:**
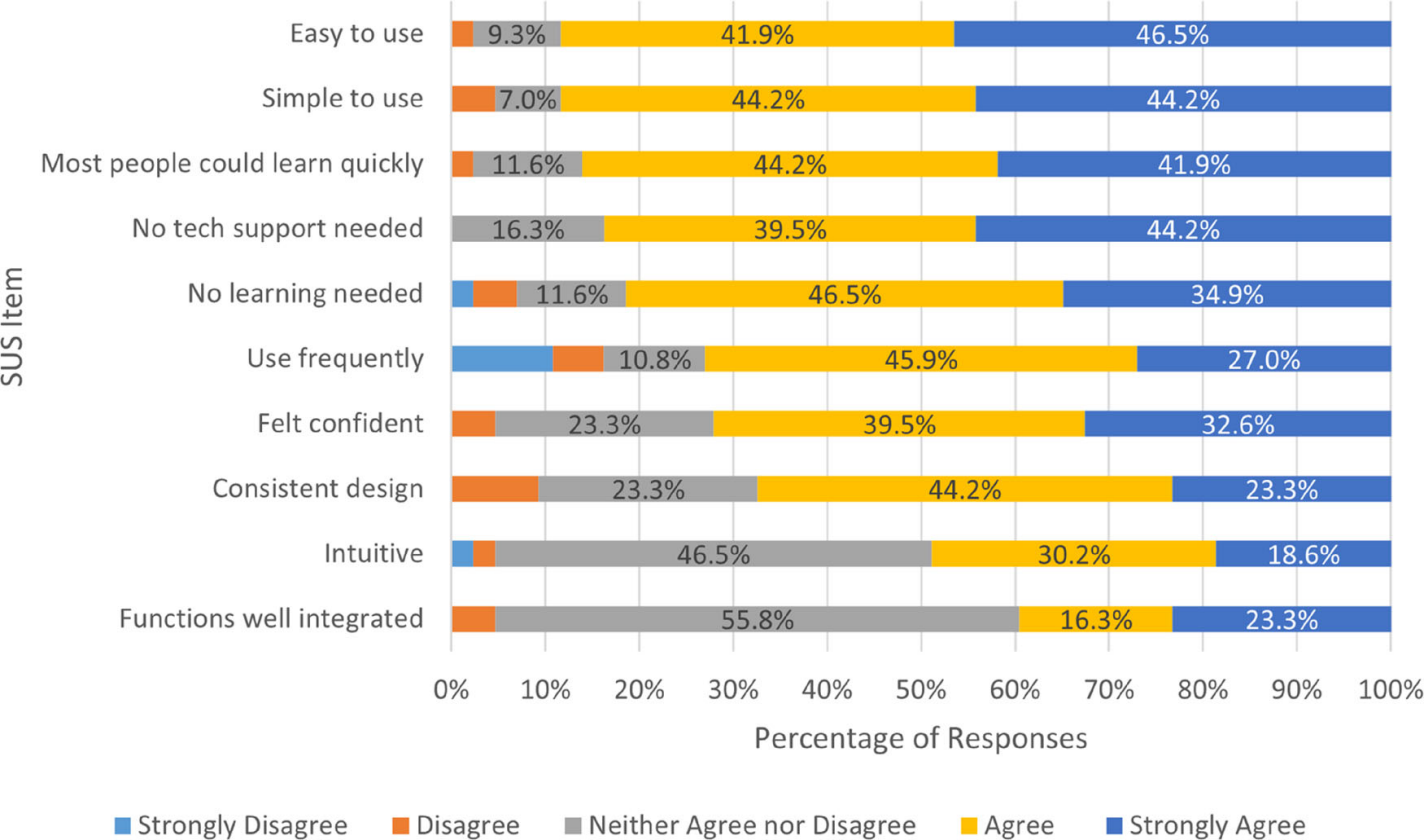
Post-Intervention Itemized Usability Scores (*n* = 43)

Across most measures (see Fig. 2), participants rated the burn alert system as highly usable (see Fig. 2). The average System Usability Score (SUS) of 74 places the system above the benchmark of 68 typically regarded as ‘good’ within the usability literature (Hyzy et al., 2022). Most considered the system easy to use (38 of 43, 88.4%) and quick to learn (37 of 43, 86%), with a similar proportion reporting that no technical support (36 of 43, 83.7%) or learning (35 of 43, 81.4%) was required. Confidence in using the system was also relatively high, with 72.1% (31 of 43) in agreement. More mixed responses were given for intuitiveness (21 of 43, 48.8% agreement) and system integration (17 of 43, 39.5% agreement), with a relatively large proportion of neutral responses on both items. These more qualified figures suggest that, while core system functions worked well, some aspects of interpretability and coherence could be developed to improve user experience.

More pointed technological influences on engagement arose in relation to the frequency and timing of alerts. Despite design efforts aimed at reducing warning fatigue, participants across engagement levels commented on the high frequency of alerts (P48, Moderate, Po-s; P47; High, Po-S). Low engagers in particular reported disregarding alert messages for this reason: ‘[If] I followed [the alerts], I feel like I’d never use my log burner’ (P26, Low/No, Po-S). Similarly, ‘[e]very day it was the same: avoid lighting your stove…there’s no point in consulting it’ (P18, Low/No, Po-S).

**Fig. 3:**
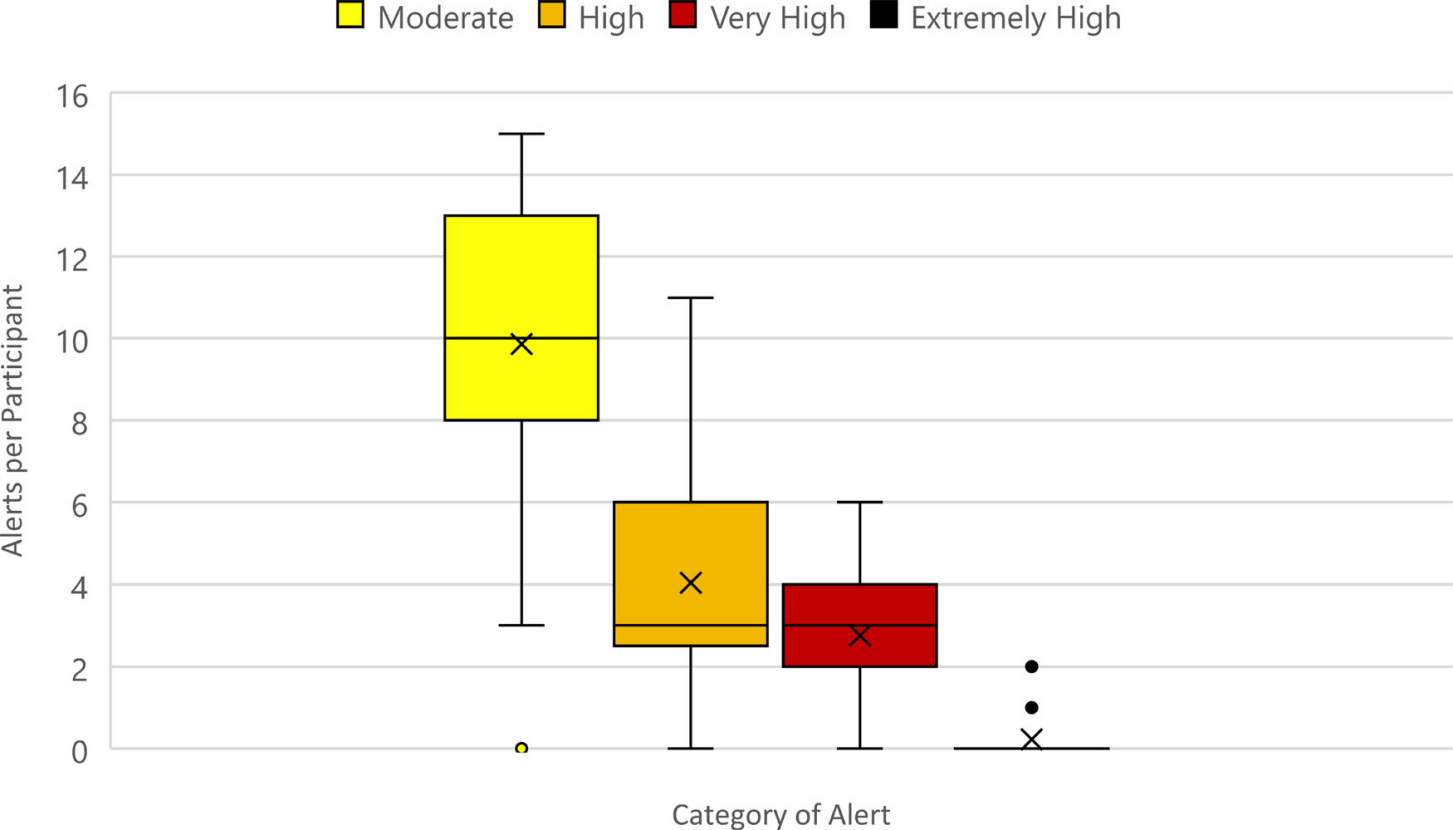
Distribution of Alerts per Participant by Level (*n* = 49)

**Fig. 4:**
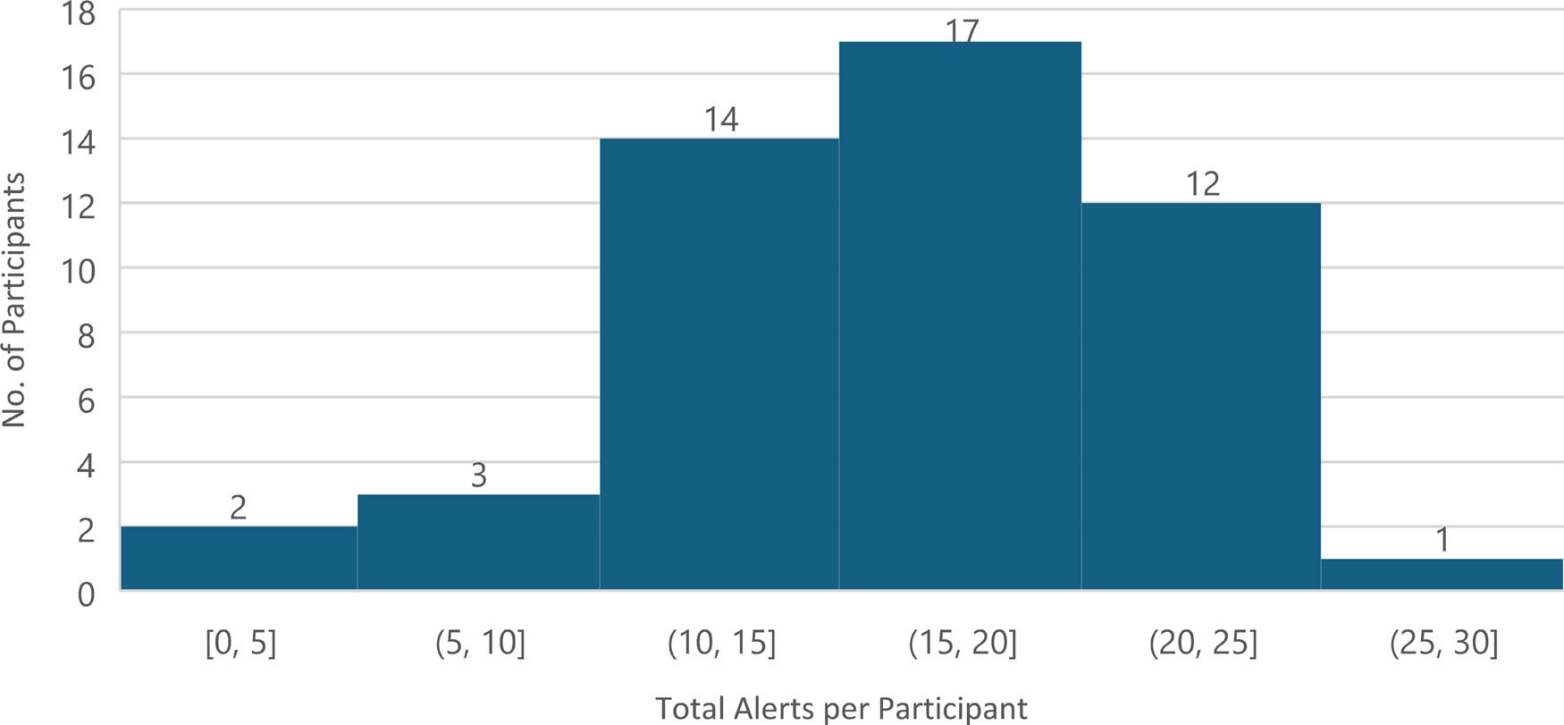
Total Alerts per Participant in the 30-day Test Period (*n* = 49)*. *Alert counts are grouped into five-alert bins to aid visual interpretation of the distribution of alerts received across participants

Table 5 provides more context on this view. Participants received alerts skewed towards the lower thresholds, meaning messages would have looked similar because they tended to cluster in the Advisory category. Moderate alerts were most commonly received, at around 10 each (IQR 8-13), whereas Very High and Extremely High alerts were comparatively rare (Fig. 3). Most participants received between 15 and 25 alerts over 30 days, with 20 of 49 (41%) participants receiving twenty or more (see Fig. 4). A second issue with the alerts concerned their being scheduled too late in the evening for some, with nine noting they had already lit their stove by 6 pm. Five of these were in the Low/No Engagement Category (Po-S) - plausibly as a direct result of the alerts arriving too late for them. A further three people in the Low/No Engagement category also noted that alerts did not coincide with the days in which they planned to burn, otherwise they would have complied. Taken together, these results indicate a need to ensure fewer push messages, prioritize higher thresholds, and consider tailoring alerts to individual household routines.

**Table 5:**
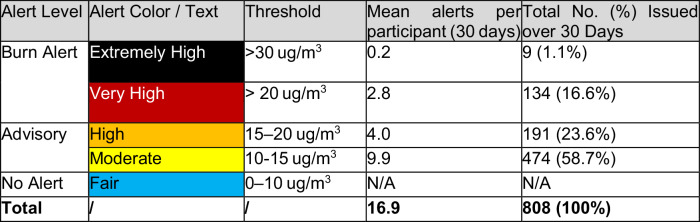
Distribution of Alerts by Level (*n* = 49)

The final technological obstacle to engagement was the absence of detail on exactly where each AQ sensor was installed. Many raised questions about data quality linked to sensor proximity, subsequently challenging their engagement:

*[Knowing sensor locations] would certainly make me trust it more because I’m thinking it’s not a generalization of Swansea…. So, [I’d] definitely not light my log burner*.

(P11, High, Int)

The burn alert animation explained that sensor density had been increased in Swansea for this study but did not give precise locations. The findings suggest the exact location of these sensors needs to be communicated, and that these locations will need to be close to users to increase trust in the data. In the absence of this, several noted relying on their senses, which often contradicted alerts (P49, Low/No; P39, Moderate, Int). As P11 (High, Int) explained, ‘[c]an’t see it, can’t smell it – it doesn’t exist. So, to be told that the air quality in my postcode is poor, I’m thinking, well it must be a monitor somewhere in my postcode…but is it? I don’t know’.

#### Aesthetics and Economics

Consistent with findings elsewhere (Kantar, 2020; Cupples et al., 2007), co-existing reasons were given for burning. Among baseline survey respondents (44 of 49), 34 (73%) reported using their stove to heat the home and 22 (50%) to save on energy costs. Notably, 17 (39%) selected both reasons, illustrating a coupling of warmth and affordability. However, when asked for their priority reason for burning, aesthetics dominated (17 of 44, 39%), followed by ‘saving on energy bills’ (14 of 44, 32%) and ‘heating the home’ (11 of 44, 25%). This highlights that while burning is motivated by multiple factors, aesthetic appeal may continue to sustain burning behaviors even in the event of declining energy costs or improved household finances.

Cost-related influences cut across the engagement spectrum, though the justification of financial necessity became more explicit as engagement became less consistent. These tended to frame burning in financial terms: ‘every time I get my gas and electric bill, I think I’m going to start using my burner more’ (P25, Low/No, Po-S; P30, Low/No, Int; P45, Low/No, Po-S). By contrast, those in the High-engagement category appeared to have more flexibility to use their central heating. Several of these described burning as a ‘luxury’ (P7, High, Int; P34, High, Po-S), and reported feeling more able to use central heating on receipt of an alert. Here, there was greater recourse to available household finances. P16 (High, Int) noted being in a ‘fortunate position’ where they could ‘afford to pay our bills’. This afforded greater flexibility: ‘I’ve got gas central heating, we’ve got heated blankets…It’s not like I would have actually frozen to death if I didn’t use it’ (P9, High, Int).

Relatedly, many participants reported being able to access wood locally for very little or no cost. Low-cost fuel options, including salvaged or free wood, were burned by 55% (24 of 49) of participants. As reflected in the qualitative data: ‘I don’t pay for the wood, I’ve got a friend who’s got a forest… every year he delivers me a pile’ (P41, High, Int). Once woodpiles are stocked and readily available, perceptions of cost are lower than alternative heating methods: ‘[with] the abundance of wood in my store outside, for me it’s a no-brainer… it’s almost like free heating’ (P1, Low/No, Int). In the context of energy prices perceived as high, the availability of low-cost fuels further positions burning as a cost-effective option.

The initial cost of purchasing and installing a stove also affected alert engagement as burning was seen as a way to recover that initial outlay. Mirroring wider evidence of households turning to wood burning in response to rising energy prices (Price-Allison et al., 2023), participants across the engagement spectrum noted that stopping burning entirely was unfeasible because of this sunk cost:

*I’ve just spent a good few thousand installing this burner and I haven’t yet kind of reached net zero…in terms of recouping the cost yet… I’m definitely, for that reason, not going to stop burning completely*.

(P20, High, Int)

*Even if you know about the impact of pollution caused by them, you’ve spent over £1000 installing one. You’re going to just stop using it?*

(P40, Low/No, Int)

Taken together, these cost-related justifications cannot be explained solely by reference to income. Participant occupations and qualifications varied, and burning cut across these socio-economic differences as a perceived means of reducing household costs. Participants were predominantly employed in the education sector (32%, 15 of 49) and health services (19%, 9 of 49), with additional representation from public administration (9%, 4 of 49). Participants were also highly educated; 85% (41 of 49) held undergraduate and postgraduate degrees, while 15% (7 of 49) reported vocational, college or secondary education. Most were also homeowners (96%, 45 of 49). Yet, burning was widely framed as one of the few cost-saving options available in a period of rising living expenses, where food, energy, and mortgage costs were seen to be increasing. In this context, the ability to offset heating costs through wood burning was seen as a tangible, controllable action, even among those otherwise relatively financially secure.

#### Information Environment

The information environment surrounding domestic burning shaped how participants understood their practices and, in turn, responded to alerts. For some, inconsistent avoidance was justified through a combination of perceived existing compliance and environmental discounting. Participants described feeling unfairly targeted by alert messages because they believed their burning was environmentally acceptable. This perception was grounded in their reading of ‘authorized’ appliance labels such as ‘EcoDesign’, ‘ClearSkies’, and ‘Defra-approved’. Rather than being understood as minimum technical standards, these were interpreted as evidence of their environmentally responsible burning:

*We installed it thinking it was low carbon… we were very keen on reducing our carbon impact*.

(P46, High, Int)

*They’re so easily available to buy… there’s no restrictions to make people think, oh this could potentially be a negative thing*.

(P34, Moderate, Int)

*There’s a huge amount of fear, uncertainty and doubt… people see the labels and think they’re fine*.

(P49, Low/No, Int)

For some, this label-based assurance allowed for environmental discounting, where use of ‘authorized’ appliances was seen to offset the need to comply with alerts. As P2 (Low/No, Int) explained, ‘I’ve got an eco-burner…I like to feel that I’ve done everything that was expected of me, to allow me to burn wood without worrying about it’. Similarly, ‘[i]f I am burning cleaner than 98% of those with a wood burner, why should I stop?’ (P49, Low/No, Po-S). Such interpretations reflect a broader tendency, evidenced elsewhere (Heydon & Chakraborty, 2022), where technical standards are taken to indicate environmental credentials extending beyond AQ.

**Fig. 5:**
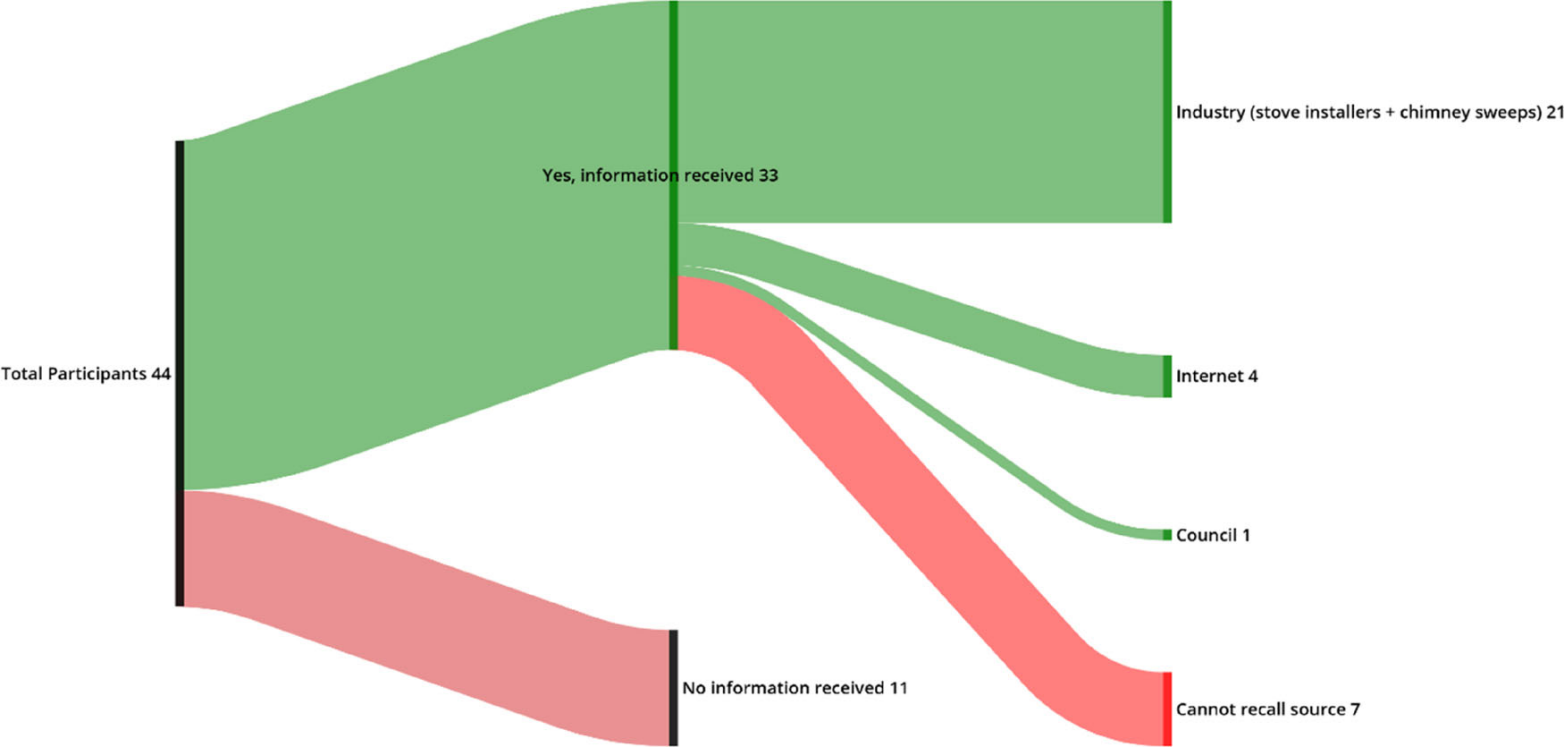
Sources for Guidance on Burning

These interpretations may be related to the limited and predominantly commercial origins of information about burning (see Fig. 5). Of the 44 people answering this question, baseline survey data indicated that 25% (11 of 44) had ‘never’ received guidance, 66% (29 of 44) a ‘moderate’ amount, and 9% (4 of 44) a ‘great deal’. Of the 33 who had received information, the dominant source was industry (21 of 33), while only one participant recalled receiving information from government. Awareness of smoke control regulations was also low. Among baseline respondents (43 of 49), two lived in an SCA but almost half (20 of 43, 46.5%) did not know (see Table 3). As (P34, Moderate, Int) noted, ‘we used to have smoke control areas. Are they still a thing?’, and (P40, Never, Int), ‘I thought it was just for coal back in the day. No-one tells you if it applies to wood burners’. Taken together, broad interpretations of ‘authorized’ appliance labels, an absence of independent information sources beyond those with a commercial interest, and limited regulatory awareness, combined to influence alert engagement. These therefore indicate potential avenues for future policy action.

#### Social Responsibility and Self-Efficacy

Health concerns were a significant motivator for those more consistently complying with alerts. Eleven of the 49 (23%) participants reported a health vulnerability in the household, six of whom were high engagers. Yet concern extended more broadly, with almost half of high engagers (10 of 24, 42%) citing health impacts as a factor underpinning compliance. For some, this derived from direct personal or household experiences of worsened symptoms following lighting (P29, High, Int; P9, High, Int). For others, health concerns extended to the wellbeing of others, with compliance justified because they had ‘no wish to add to my neighbors’ burden or exposure’ [sic] (P30, High, Po-S):

*The thought of me sending stuff out into the atmosphere for other kids in the street that might have respiratory conditions, or adults that have them – it definitely did make me rethink* (P7, High, Int).

*Some of my neighbors really aren’t that fit and healthy, some have kids, some are older, some are really poorly* (P31, High, Int).

For higher engagers, compliance was therefore often framed as a form of social responsibility rooted in health concerns. However, responses were tempered by varying perceptions of self-efficacy in the context of other pollution sources. Local industry (especially the Port Talbot steelworks), vehicles, wildfires, and other domestic burners, were all cited as persistent and under-regulated contributors to poor AQ that perhaps excused individual burning. While higher engagers saw their own actions to curtail or manage burning as still meaningful in this context (P9, High, Int), those in other categories expressed less certainty:

*I was more likely to follow the advice when the alert was at Medium, as it felt more like we could make a difference*.

(P14, Moderate, Po-S).

*I don’t know, really, how much difference I would make. I’m only one person, one household*.

(P28, Moderate, Int).

*My road is pretty heavily trafficked by motor vehicles, and I doubt lighting or not lighting my stove is going to be a game changer*.

(P49, Low/No, Po-S).

Taken together, health concerns and a sense of social responsibility supported greater engagement, particularly among high engagers. Yet varying perceptions of self-efficacy, and forms of environmental discounting, shaped how far these motivations translated into consistent compliance.

## Discussion

This pilot study examined whether a voluntary, hyper-local burn alert system could supplement existing domestic burning controls by discouraging lighting during high PM2.5 episodes, improving AQ literacy, and reducing emissions at source. Among participants who engaged with the system, alerts were associated with substantial reductions in reported burning and improvements in AQ literacy over the four-week trial. While causal inference is limited, these findings suggest that behaviorally informed alerts combining local AQ data, health framing and clear recommendations to avoid burning can support short-term changes in emission-producing practices. This reinforces evidence on the effectiveness of concise and actionable messaging in AQ communications (Riley et al., 2021; Sunter et al., forthcoming).

These effects were, however, unevenly distributed. To understand how the system influenced burning behavior and AQ literacy, the IBM-WASH framework was used to analyze engagement. While technological factors such as alert design and timing acted as important enablers, engagement was also shaped by contextual and psychosocial influences. These included energy costs, household routines, health vulnerabilities, trust in the intervention, and the signaling effects of ‘authorized’ appliance labeling. Taken together, the results indicate that while voluntary burn alerts can dissuade burning and improve AQ literacy among users, uptake and sustained engagement are strongly influenced by factors outside of the intervention itself.

A central insight from this study is therefore that, although burn alerts may reduce emissions among engaged users, population-level effectiveness is likely to be constrained by limited uptake. As with other WASH and household AQ interventions, impact scales with participation (Clasen & Smith, 2019). Low recruitment relative to the estimated burning population of Swansea suggests that wider contextual factors restrict engagement. AQ remains low on the public agenda, (Defra, 2025a: 52), willingness to reduce domestic burning is limited (Defra, 2025a: 58), and support for additional regulation is weak (Defra, 2025a: 61). These constraints are compounded by persistently high energy costs (Pittaway & Try, 2025), with domestic burning often perceived as a strategy for managing household expenditure (Defra, 2024; Price-Allison et al., 2023). In this context, low uptake of a system that discourages stove use is perhaps unsurprising. It may also help to explain why burn alert schemes elsewhere rely on enforcement mechanisms to sustain participation (see Heydon et al., 2024: 773).

Encouraging wider uptake is further challenged by the nature of the behavior targeted. Many AQ interventions focus on behaviors aimed at reducing *exposure*, where the social acceptability of the underlying activity is left unquestioned. By contrast, burn alerts directly target *emission-producing* behaviors. By linking harm reduction to use reduction, they implicitly challenge the widely accepted framing of domestic burning as a harmless activity embedded in routine practice. This tension is reinforced by a policy landscape that continues to legitimate stove use through ‘authorized’ appliance labels and SCA exemptions. As AQ standards tighten and awareness of health impacts grows, other interventions that target equally normalised behaviors could become contested in similar ways (Air Quality Expert Group, 2024).

To mitigate these tensions and improve uptake and engagement, the findings indicate that effective voluntary burn alert systems would benefit from interventions at multiple levels. Drawing on the IBM-WASH framework, these include strengthening public information and advertising standards at the societal level; incorporating participatory design approaches at the community level (see Cibin et al., 2025); and improving system usability, relevance and feedback at the household and individual level. Table 6 provides a structured summary of these recommendations, linking proposed actions to IBM-WASH dimensions and the contextual, psychosocial and technological factors shaping engagement.

**Table 6:** Recommendations for Improving Burn Alert Uptake, Engagement and Outcomes

Action	Target Level	Relevant Dimensions	Purpose
Formally introduce burn alert system as voluntary regulation, widely publish public health justifications, threshold logic and local authority updates, consider integration with SCA regulations	Societal/Structural	Contextual Psychosocial	Emphasizes health-based trigger, reduces perceived unfairness, normalizes episode response, increases uptake
Provide financial support to low-income households for home heating, introduce installation restrictions	Societal/Structural	Contextual	Removes economic constraint to complying with burn alert advice, removes future prevalence of sunk-cost justification for burning/non-compliance
Tighten advertising standards around ‘authorized appliances’, improve public AQ literacy around air quality, domestic burning and ‘authorized labels’	Societal/Structural	Contextual Psychosocial	Reduces moral licensing, improves AQ literacy, reframes idea that ‘authorized’ label provides permission to burn
Introduce point-of-sale/installer sign-up prompts to alert system	Societal/Structural	Contextual Psychosocial	Targets enrollment at new users, normalizes alert system as part of burning practice, improves AQ literacy, introduces alternative information source
Include fuel guidance on web interface/future apps, local authority widely publicize system to users and non-users	Community	Contextual Technological Psychosocial	Reframes the burning of free/cheap fuel as without cost, increases awareness beyond new users, improves wider AQ literacy
Emphasize health-salience, include endorsements from local groups (e.g. schools, health charities), include local groups in communications and intervention design	Community	Contextual Technological Psychosocial	Increases awareness of impact on neighbors/local people, improves location-specific messaging, increases participatory nature of intervention
Maintain exemptions where no viable heating alternative exists, consider advisory-only alerts during severe cold, signpost assistance in app (insulation heating subsidies, temporary bill supports)	Interpersonal/Household	Contextual Technological Psychosocial	Equity safeguards, acknowledges cost-of-living and sunk-cost constraints
Show ‘nearest monitor’ distance in alert text, publish siting criteria and updated sensor map, show area participation rate, integrate with alerts targeting suite of behaviors	Community	Technological Psychosocial	Proximity and transparency raise trust in accuracy, reduces norm ambiguity, expands scope of uptake
Forecast-based alerts and personalized alert timings	Interpersonal/Household	Technological Psychosocial	Disrupts pre-lighting decision, intercepts household routine
Emphasize effect of each avoided burn (e.g. ‘skipping once avoids approx. X hours of emissions’)	Individual	Technological Psychosocial	Raises self-efficacy, quantifies effects of behavior
Raise lowest alert threshold, per-user frequency cap, more dynamic subject lines/message variation/positive reinforcement, provide opportunities for further information	Individual	Technological Psychosocial	Manages alert fatigue, rewards engagement

### Study Limitations

Several limitations should be considered when interpreting these findings. First, the study relied primarily on self-reported survey data to assess compliance, attitudes, and behavior change. Although these were supplemented by open-text responses and follow-up interviews to improve internal validity, the influence of social desirability and recall biases cannot be excluded. Participants also entered the trial with varying levels of AQ knowledge. While AQ literacy improved on average, differences in prior understanding may have shaped how alerts were interpreted and acted upon.

Second, ambient AQ concentrations during the trial were influenced by factors beyond domestic burning, including meteorological conditions (e.g. wind speed and direction, boundary layer height and precipitation), housing characteristics (such as proximity to roads), and background pollution sources (including traffic, industrial emissions and transboundary transport). Meteorological normalization techniques were considered but not implemented, as the primary aim was to assess behavioral responses to alerts rather than attribute concentration changes to specific emission sources.

Third, the sample was small relative to the estimated burning population of Swansea (see Lewis, 2023). While this reflects the voluntary nature of the system, approximating likely uptake under similar real-world conditions, it limits the generalizability of the findings. The four-week trial period also restricts conclusions about the durability of behavioral change and AQ literacy gains over time. Longer-term evaluation would be helpful to assess sustained engagement and potential cumulative effects.

## Conclusion

This pilot study demonstrates that a voluntary, hyper-local burn alert system can reduce domestic burning and improve AQ literacy among participating households. However, it also shows that engagement with the system is uneven, and shaped by wider contextual, psychosocial and technological factors. As an exploratory intervention, the study does not provide population-level estimates of impact, but offers insight into the mechanisms, constraints and opportunities associated with voluntary approaches to domestic burning control. The findings suggest that voluntary alerts are best understood as an episode-specific, decision-support intervention, rather than a stand-alone solution to structurally embedded sources of domestic emissions.

Within these limits, the findings suggest that burn alerts could be most effective when embedded within broader strategies aimed at addressing household energy pressures, increasing low public AQ literacy, and improving trust in interventions. Future research should assess whether such complementary measures increase uptake, examine the durability of behavioral and literacy effects over longer periods, and determine whether there would be utility in burn alerts being incorporated into a modified regulatory regime.

## Supplementary information

SI 1-Pre-Intervention Survey

SI 2-Post-Intervention Survey

## Data Availability

The data that support the findings of this study are available from the authors upon reasonable request.
