# Correction to: Reducing Domestic Wood Burning through Voluntary Air Quality Alerts: An IBM-WASH Evaluation of a Pilot Intervention in Wales

**DOI:** 10.1007/s00267-026-02519-9

**Published:** 2026-06-06

**Authors:** James Heydon, Menna Price, Ian Walker, Paul Lewis, Rohit Chakraborty, Caitlin Bunce, Kori Sunter

**Affiliations:** 1 University of Nottingham, Nottingham, UK; 2 Swansea University, Swansea, UK; 3 Swansea University, Raven Delta Group, Swansea, UK; 4 UK Health Security Agency, London, UK

Correction to: Environmental Management

10.1007/s00267-026-02463-8

In this article there was an omission in the Author contributions and Funding statement sections. The original article has been corrected.

