# Supplementary material for: Reducing Domestic Wood Burning through Voluntary Air Quality Alerts: An IBM-WASH Evaluation of a Pilot Intervention in Wales
Source: Environ Manage. 2026 Apr 22;76(5):171. doi: 10.1007/s00267-026-02463-8 (PMC13102875; doi:10.1007/s00267-026-02463-8)
Supplement: Supplementary file 1 — SI 1-Pre-Intervention Survey [file 267_2026_2463_MOESM1_ESM.docx]

Supplementary Information I)

Burn Alert Pre-Intervention Survey

Survey Flow

Standard: Information and Consent (9 Questions)

Standard: Demographics (9 Questions)

Standard: Use and Behaviours (12 Questions)

Standard: Wood Burner Use Air Quality Literacy (13 Questions)

Standard: Debrief (1 Question)

| Page Break |  |
| --- | --- |

Start of Block: Information and Consent

Captcha Please confirm you are human by ticking the box below:

**Participant Information Sheet**

PI confirmation I have read and understood the participant information sheet and agree to complete the survey on that basis.

- I agree (continue to survey) (1)

Skip To: End of Survey If PI confirmation != 1

| Page Break |  |
| --- | --- |

| 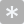 |
| --- |

Device Participation in this study requires you to have both a wood burner **and** an alternative source of heat. Please confirm you have (tick) both of the following:

- A wood burner (e.g. log burning stove, open fireplace, etc.) (1)
- An alternative heat source (e.g. central heating, gas fire, electric fire, etc.) (2)

Skip To: End of Survey If Condition: Selected Count Is Equal to 1. Skip To: End of Survey.

| Page Break |  |
| --- | --- |

| 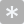 |
| --- |

Email What is your email address? We will use this to communicate and match this survey to your second survey

________________________________________________________________

- **Consent form**

End of Block: Information and Consent

Start of Block: Demographics

2 How would you like to receive air quality alerts? This will not be shared with anyone. We will use this information to send you a daily alert of the air quality around your home. We will also use this information to match the two surveys.

- Email (you have provided this already) (1)
- SMS text message, please enter number: (4) __________________________________________________

| 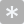 |
| --- |

3 What is your date of birth? Please use dd/mm/yyyy format. (We will use this information to match the two surveys)

________________________________________________________________

4 What gender do you identify as?

- Female (1)
- Male (2)
- Transgender Female (3)
- Transgender Male (4)
- Gender Variant/Non Conforming (5)
- Prefer not to say (6)
- Other (please specify) (7) __________________________________________________

5 What is your ethnic group?

- Asian (1)
- Asian Welsh (2)
- Black (3)
- Black British (4)
- Black Welsh (5)
- Caribbean (6)
- African (7)
- Mixed (8)
- Multiple ethnic groups (9)
- White (10)
- Other ethnic group (please specify) (11) __________________________________________________

6 What is your job title/role? (Type answer next to your sector). If sector is not labelled, please enter sector and job title in 'Other'

- Retail or Food/Beverage Services (1) __________________________________________________
- Education (2) __________________________________________________
- Construction (3) __________________________________________________
- Health services (4) __________________________________________________
- Trade, Buisness or Financial Services (5) __________________________________________________
- Computer programming or Software Services (6) __________________________________________________
- Public Administration and Defence or Politics (7) __________________________________________________
- Transport (8) __________________________________________________
- Entertainment (9) __________________________________________________
- Self-employed (10) __________________________________________________
- Unemployed (11)
- Other (12) __________________________________________________

7 What is the highest level of education you have completed?

- Some Primary (1)
- Completed Primary School (2)
- Some Secondary (3)
- Completed Secondary School (4)
- Vocational or Similar (11)
- Some Sixth Form/College (5)
- All Sixth Form/College (6)
- Some University but no degree (7)
- University Bachelors Degree (8)
- Graduate or professional degree (MA, MS, MBA, PhD, JD, MD, DDS) (9)
- Prefer not to say (10)
- Other (please specify) (12) __________________________________________________

8 How many children (under 18) live in your home?

- 0 (1)
- 1 (2)
- 2 (3)
- 3 (4)
- 4 (5)
- 5 (6)
- 6 (7)
- 7+ (8)

9 Does anybody in your household have a respiratory condition (e.g. asthma, cystic fibrosis, COPD)? (Tick all that apply)

- No (1)
- Me (2)
- Partner (3)
- Parent (4)
- Child/Children (5)
- Other (6)

10 Are you a homeowner?

- Yes (1)
- No (2)

End of Block: Demographics

Start of Block: Use and Behaviours

11 What is the make and model of your wood burner (or the one you use most frequently)?

- Enter (3) __________________________________________________
- I don't know (4)

12 What year was your wood burner installed?

- Enter (3) __________________________________________________
- I don't know (4)

13 When was your wood burner last serviced?

- Enter (3) __________________________________________________
- I don't know (4)
- Never (5)

14 Do you live in a smoke control area?

- Yes (1)
- No (2)
- I don't know (3)

| 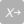 |
| --- |

15 Why do you mostly use a log burner? (Tick all that apply)

- Heat my home (1)
- Because it's cosy (2)
- Helps me to save on energy bills (3)
- Because it's environmentally friendly (Please explain how) (4) __________________________________________________
- Other (5) __________________________________________________

| 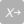 |
| --- |

16 Of the reasons given, which of the most important to you? (Select one)

- Heat my home (1)
- Because it's cosy (2)
- Helps me to save on energy bills (3)
- Because it's environmentally friendly (4)
- Other (5) __________________________________________________

| 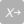 |
| --- |

17  How frequently do you use a wood burner during the colder months?

- Everyday (4)
- 2-3 times a week (3)
- 2-3 times a month (2)
- Once a month (1)
- Never (0)

18 Typically, for how many hours is your wood burner usually lit?

- Less than 1 hour (1)
- 1-2 hours (2)
- 2-3 hours (3)
- 4-5 hours (4)
- 5 hours or more (5)

19 What do you typically burn in your stove?

- Store-bought wood (e.g. logs, pellets, briquettes, wood chips) (2)
- Salvaged wood (e.g. scrap wood, felled trees, garden waste) (3)
- House coal (4)
- Smokeless coal (5)
- Other. Please detail what material(s): (7) __________________________________________________

20 Where do you get your wood from? (tick all that apply)

- Collect fallen wood (e.g. from trees) (1)
- Collect scrap wood (e.g. old pallet pieces) (2)
- Buy dried wood from a physical shop, farm, petrol station, etc (3)
- Buy dried wood from an online shop (4)
- N/A - I do not burn wood (e.g. use coal only) (5)

| 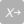 |
| --- |

21 Have you received information or guidance on good practice when using a wood burner?

- No, never (1)
- Yes, a moderate amount (2)
- Yes, a great deal (3)

Skip To: End of Block If 21 = 1

21b Where did you receive this information?

- Can't recall (1)
- Enter details: (2) __________________________________________________

End of Block: Use and Behaviours

Start of Block: Wood Burner Use Air Quality Literacy

Literacy explainer This section asks about your understanding of wood burner use. Please answer the questions to the best of your current knowledge.

| 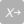 |
| --- |

22 When burned in a home log burner, which type of fuel releases the most particles from the chimney?

- Wet Wood (0)
- Seasoned Wood (-1)
- Waste Wood (1)

| 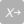 |
| --- |

23 Which of these heating methods will lead to poorest indoor air for the person using it?

- Electric radiator (-1)
- Gas Heater (0)
- Wood Burner (1)

| 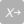 |
| --- |

24 Which of these is NOT a health risk resulting from burning fuel?

- Hydrogen (1)
- Fine particles (0)
- Nitrogen Dioxide (-1)

| 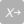 |
| --- |

25 Is it more harmful to your health to breathe small particles of smoke or large particles of smoke?

- Small (1)
- Large (0)
- They are equally harmful (-1)

| 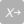 |
| --- |

26 What type of emissions are commonly associated with the use of wood burners?

- Carbon dioxide (CO2) only (0)
- Particulate Matter (PM), Carbon Monoxide (CO), and Volatile organic compounds (VOCS) (1)
- Nitrogen Oxide (NOx) and Sulphur Dioxide (SO2) (-1)

| 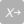 |
| --- |

27 How does the type of wood used in a wood burner affect air pollution?

- Hardwood produces fewer emissions than softwood (1)
- Softwood produces fewer emissions than hardwood (0)
- The impact is the same regardless of the wood (-1)

| 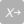 |
| --- |

28 What practice is recommended for reducing air pollution from wood burners?

- Burning wet or unseasoned wood (0)
- Restricting ventilation to increase heat output (-1)
- Regularly cleaning the chimney to remove creosote (1)

| 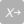 |
| --- |

29 How can exposure to wood burner emissions impact human health?

- No health risks are associated with wood burner emissions (0)
- It specifically causes respiratory problems (Lung irritation and Asthma) (-1)
- It causes a range of health problems (1)

| 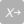 |
| --- |

30 Which of the following is considered a cleaner alternative to traditional wood burners?

- Open fireplace (0)
- Coal stove (-1)
- Pellet stove (1)

| 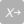 |
| --- |

31 Which of these bundles of firewood will burn the cleanest if used on a log burner?

- Wood with 40% moisture content (0)
- Wood with 15% moisture content (1)
- Wood with 30% moisture content (-1)

| 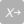 |
| --- |

32 Which of these smoke directions would be the most dangerous for people walking around? A     B C

- A - Vertical (0)
- B - Horizontal (1)
- C - Diagonal (-1)

| 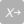 |
| --- |

33 Which of these actions would make it safest to burn wood indoors?

- Opening an air vent (0)
- Opening a window (1)
- Turning on a fan (-1)

End of Block: Wood Burner Use Air Quality Literacy

Start of Block: Debrief

Debrief

End of Block: Debrief
