# Supplementary material for: Reducing Domestic Wood Burning through Voluntary Air Quality Alerts: An IBM-WASH Evaluation of a Pilot Intervention in Wales
Source: Environ Manage. 2026 Apr 22;76(5):171. doi: 10.1007/s00267-026-02463-8 (PMC13102875; doi:10.1007/s00267-026-02463-8)
Supplement: Supplementary file 2 — SI 2-Post-Intervention Survey [file 267_2026_2463_MOESM2_ESM.docx]

Supplementary Information II)

Burn Alert Post-Intervention Survey

Survey Flow

Standard: Survey match (4 Questions)

Standard: Burn Alert Engagement (24 Questions)

Standard: Burn Alert Usability (3 Questions)

Standard: Burn Alert Development (1 Question)

Standard: Wood Burner Use Air Quality Literacy (13 Questions)

Standard: Follow-up (2 Questions)

Standard: Debrief (1 Question)

| Page Break |  |
| --- | --- |

Start of Block: Survey match

Q263 What is your postcode? This will not be shared with anyone. We will use this information to match the two surveys.

________________________________________________________________

Display this question:

If If False

2 What is your email address? We will also use this information to match your two surveys.

________________________________________________________________

| 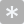 |
| --- |

2a What is your date of birth? Please use dd/mm/yyyy format. We will also use this information to match the two surveys

________________________________________________________________

End of Block: Survey match

Start of Block: Burn Alert Engagement

3 What was the most frequent way you engaged with the Burn Alert advice? (Drag and drop)

______ I would wait for the SMS alerts (1)

______ I would wait for the email alerts (2)

______ I checked the Burn Alert website on my smartphone (3)

______ I checked the Burn Alert website on my desktop/laptop/tablet computer (4)

| Page Break |  |
| --- | --- |

4 Did you receive one or more alerts (including yellow, amber, red, black) over the last four weeks?

- Yes (1)
- No (2)

Skip To: 12 If 4 = 2

5 How often did you follow the Burn Alert advice not to light your stove?

- Never (1)
- Rarely (2)
- Sometimes (3)
- Often (4)
- Always (5)

Display this question:

If 5 = 1

exp never. Please explain why you never followed the Burn Alert advice

________________________________________________________________

________________________________________________________________

________________________________________________________________

________________________________________________________________

________________________________________________________________

Display this question:

If 5 = 2

exp rarely. Please explain why you rarely followed the Burn Alert advice

________________________________________________________________

________________________________________________________________

________________________________________________________________

________________________________________________________________

________________________________________________________________

Display this question:

If 5 = 3

exp sometimes. Please explain why you sometimes followed the Burn Alert advice

________________________________________________________________

________________________________________________________________

________________________________________________________________

________________________________________________________________

________________________________________________________________

Display this question:

If 5 = 4

exp often. Please explain why you often followed the Burn Alert advice

________________________________________________________________

________________________________________________________________

________________________________________________________________

________________________________________________________________

________________________________________________________________

Display this question:

If 5 = 5

exp always Please explain why you always followed the Burn Alert advice

________________________________________________________________

________________________________________________________________

________________________________________________________________

________________________________________________________________

________________________________________________________________

| Page Break |  |
| --- | --- |

Display this question:

If 5 = 2

And 5 = 3

And 5 = 4

And 5 = 5

6 Roughly, how many times over the last four weeks did you decide not to light your stove because of Burn Alert advice?

▼ 0 (33) ... 30 + (3)

Display this question:

If 5 = 2

Or 5 = 3

Or 5 = 4

Or 5 = 1

7 Did the Burn Alert affect how long you would light your stove for?

- Yes (1)
- No (2)
- Sometimes (3)

Display this question:

If 7 = 2

8 Please explain why the Burn Alert did not affect how long you lit your stove for

________________________________________________________________

Display this question:

If 7 = 1

Or 7 = 3

9 How did the Burn Alert affect how long the stove was lit?

- I would use the stove for a shorter period of time (1)
- I would use the stove for a longer period of time (2)

Display this question:

If 9 = 1

10 Roughly, how many hours less than normal did you light your stove each time?

________________________________________________________________

Display this question:

If 9 = 2

11 Roughly, how many hours more than normal did you light your stove each time?

________________________________________________________________

| Page Break |  |
| --- | --- |

12 How often did the Burn Alert encourage you to burn when you would not have otherwise?

- Never (1)
- Rarely (2)
- Sometimes (3)
- Often (4)
- Always (5)
- I don't know (7)

Display this question:

If 12 = 1

Or 12 = 2

Or 12 = 3

Or 12 = 4

Or 12 = 5

12a Please explain your answer:

________________________________________________________________

13 If it continued to exist beyond this study period, would you continue to use the Burn Alert in future?

- Yes (1)
- No (2)
- Maybe (3)

Display this question:

If 13 = 1

Or 13 = 2

Or 13 = 3

13a Please explain your answer:

________________________________________________________________

14 How frequently have you used your wood burner over the last month while taking part in this trial?

- Everyday (1)
- 2-3 times a week (2)
- 2-3 times a month (3)
- Once a month (4)
- Never (5)

Skip To: End of Block If 14 = 5

| Page Break |  |
| --- | --- |

15 Over the last month, for how many hours was your wood burner lit on each occasion you used it?

- Less than 1 hour (1)
- 1-2 hours (2)
- 2-3 hours (3)
- 3-4 hours (4)
- 4-5 hours (5)
- 5 hours or more (6)

16 Over the last month, what have you typically burned in your stove? (tick all that apply)

- Store-bought wood (e.g. logs, pellets, briquettes, wood chips) (2)
- Salvaged wood (e.g. scrap wood, felled trees, garden waste) (3)
- House coal (4)
- Smokeless coal (5)
- Other. Please detail what material(s): (7) __________________________________________________

Display this question:

If 16 = 2

Or 16 = 3

17 Over the last month, where have you got your wood from? (tick all that apply)

- Collect fallen wood (e.g. from trees) (1)
- Collect scrap wood (e.g. old pallet pieces) (2)
- Buy dried wood from a physical shop, farm, petrol station, etc (3)
- Buy dried wood from an online shop (4)

18 Over the last month, have you received any information or guidance on good practice when using a wood burning?

- No, never (1)
- Yes, a moderate amount (2)
- Yes, a great deal (3)

Display this question:

If 18 = 2

Or 18 = 3

19 Where did you get this information?

________________________________________________________________

End of Block: Burn Alert Engagement

Start of Block: Burn Alert Usability

20-33 Burn Alert Usability

|  | Strongly disagree (1) | Disagree (2) | Neither agree nor disagree (3) | Agree (4) | Strongly agree (5) |
| --- | --- | --- | --- | --- | --- |
| I think that I would like to use the Burn Alert frequently (1) |  |  |  |  |  |
| I found the Burn Alert simple to use (2) |  |  |  |  |  |
| I thought the Burn Alert was easy to use (3) |  |  |  |  |  |
| I think I could use the Burn Alert without the need for technical support (4) |  |  |  |  |  |
| I found the various functions of the Burn Alert were well integrated (14) |  |  |  |  |  |
| I thought there was a lot of consistency in the Burn Alert (15) |  |  |  |  |  |
| I imagine that most people would learn to use the Burn Alert quickly (16) |  |  |  |  |  |
| I found the Burn Alert very intuitive (17) |  |  |  |  |  |
| I felt very confident using the Burn Alert (18) |  |  |  |  |  |
| I could use the Burn Alert without having to learn anything new (19) |  |  |  |  |  |

30-33 Burn Alert Usability

|  | - | | | | | - |
| --- | --- | --- | --- | --- | --- | --- |
|  | Strongly disagree (1) | Disagree (2) | Neither agree nor disagree (3) | Agree (4) | Strongly agree (5) | Please explain your answer (1) |
| The Burn Alert website communicates enough information about the health effects of stove emissions (1) |  |  |  |  |  |  |
| The Burn Alert introductory video communicates enough information about the health effects of stove emissions (2) |  |  |  |  |  |  |
| The alerts themselves communicate enough information about the health effects of stove emissions (3) |  |  |  |  |  |  |
| I trusted the air quality data on which the alerts were based (4) |  |  |  |  |  |  |

34 How do you think the Burn Alert could be improved?

________________________________________________________________

________________________________________________________________

________________________________________________________________

________________________________________________________________

________________________________________________________________

End of Block: Burn Alert Usability

Start of Block: Burn Alert Development

BAD 1-2 Burn Alert Development

|  | - | | | - |
| --- | --- | --- | --- | --- |
|  | Yes (1) | No (2) | Maybe (3) | Please explain your answer: (1) |
| Would you like to see this system rolled out across Swansea, for everybody to access? (1) |  |  |  |  |
| Would you like to see this voluntary system rolled out across Wales, for everybody to access? (2) |  |  |  |  |

End of Block: Burn Alert Development

Start of Block: Wood Burner Use Air Quality Literacy

Literacy explainer This section asks about your understanding of wood burner use. Please answer the questions to the best of your current knowledge.

| 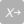 |
| --- |

22 When burned in a home log burner, which type of fuel releases the most particles from the chimney?

- Wet Wood (0)
- Seasoned Wood (-1)
- Waste Wood (1)

| 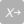 |
| --- |

23 Which of these heating methods will lead to poorest indoor air for the person using it?

- Electric radiator (-1)
- Gas Heater (0)
- Wood Burner (1)

| 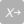 |
| --- |

24 Which of these is NOT a health risk resulting from burning fuel?

- Hydrogen (1)
- Fine particles (0)
- Nitrogen Dioxide (-1)

| 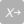 |
| --- |

25 Is it more harmful to your health to breathe small particles of smoke or large particles of smoke?

- Small (1)
- Large (0)
- They are equally harmful (-1)

| 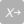 |
| --- |

26 What type of emissions are commonly associated with the use of wood burners?

- Carbon dioxide (CO2) only (0)
- Particulate Matter (PM), Carbon Monoxide (CO), and Volatile organic compounds (VOCS) (1)
- Nitrogen Oxide (NOx) and Sulphur Dioxide (SO2) (-1)

| 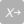 |
| --- |

27 How does the type of wood used in a wood burner affect air pollution?

- Hardwood produces fewer emissions than softwood (1)
- Softwood produces fewer emissions than hardwood (0)
- The impact is the same regardless of the wood (-1)

| 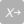 |
| --- |

28 What practice is recommended for reducing air pollution from wood burners?

- Burning wet or unseasoned wood (0)
- Restricting ventilation to increase heat output (-1)
- Regularly cleaning the chimney to remove creosote (1)

| 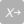 |
| --- |

29 How can exposure to wood burner emissions impact human health?

- No health risks are associated with wood burner emissions (0)
- It specifically causes respiratory problems (Lung irritation and Asthma) (-1)
- It causes a range of health problems (1)

| 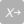 |
| --- |

30 Which of the following is considered a cleaner alternative to traditional wood burners?

- Open fireplace (0)
- Coal stove (-1)
- Pellet stove (1)

| 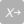 |
| --- |

31 Which of these bundles of firewood will burn the cleanest if used on a log burner?

- Wood with 40% moisture content (0)
- Wood with 15% moisture content (1)
- Wood with 30% moisture content (-1)

| 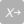 |
| --- |

32 Which of these smoke directions would be the most dangerous for people walking around? A     B C

- A - Vertical (0)
- B - Horizontal (1)
- C - Diagonal (-1)

| 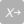 |
| --- |

33 Which of these actions would make it safest to burn wood indoors?

- Opening an air vent (0)
- Opening a window (1)
- Turning on a fan (-1)

End of Block: Wood Burner Use Air Quality Literacy

Start of Block: Follow-up

45 I am interested in taking part in a short online/telephone discussion with a member of the research team about my experiences using the burn alert system

- Yes (1)
- No (2)

46 I would like to receive any outputs/publications from this study

- Yes (1)
- No (2)

End of Block: Follow-up

Start of Block: Debrief

Debrief
**Debrief** doctor).  If you have any further questions, please contact Burnalert@Swansea.ac.uk **Please submit your survey responses below:**

End of Block: Debrief
